# Neural recordings can differentiate between spontaneously metastasizing melanomas and melanomas with low metastatic potential

**DOI:** 10.1371/journal.pone.0297281

**Published:** 2024-02-15

**Authors:** Jay Shiralkar, Tiana Anthony, Grant A. McCallum, Dominique M. Durand

**Affiliations:** Department of Biomedical Engineering, Case Western Reserve University, Cleveland, Ohio, United States of America; Rutgers University, UNITED STATES

## Abstract

Multiple studies report that melanomas are innervated tumors with sensory and sympathetic fibers where these neural fibers play crucial functional roles in tumor growth and metastasis with branch specificity. Yet there is no study which reports the direct neural recording and its pattern during in-vivo progression of the cancer. We performed daily neural recordings from male and female mice bearing orthotopic metastasizing- melanomas and melanomas with low metastatic poential, derived from B16-F10 and B16-F1 cells, respectively. Further, to explore the origins of neural activity, 6-Hydroxidopamine mediated chemical sympathectomy was performed followed by daily microneurographic recordings. We also performed the daily bioluminescent imaging to track in vivo growth of primary tumors and distant metastasis to the cranial area. Our results show that metastasizing tumors display high levels of neural activity while tumors with low metastatic potential lack it indicating that the presence of neural activity is linked to the metastasizing potential of the tumors. Moreover, the neural activity is not continuous over the tumor progression and has a sex-specific temporal patterns where males have two peaks of high neural activity while females show a single peak. The neural peak activity originated in peripheral sympathetic nerves as sympathectomy completely eliminated the peak activity in both sexes. Peak activities were highly correlated with the distant metastasis in both sexes. These results show that sympathetic neural activity is crucially involved in tumor metastasis and has sex-specific role in malignancy initiation.

## 1 Introduction

Melanoma, although it only makes up 1% of skin cancers, causes the majority of skin cancer deaths. The number of melanoma cases has been rapidly increasing in recent decades and it is becoming a significant public health issue worldwide [1]. Early detection is crucial in reducing deaths from melanoma, where exposure to ultraviolet radiation is believed to be a major cause, although genetics and family history also play a role [1]. Treatment options include immunotherapy, chemotherapy, targeted drug therapy, radiation therapy, and surgery, but all have severe side effects. The 5-year overall survival of patients with metastasizing cutaneous melanoma remains between 5% and 19% [2]. Melanoma is more common in older individuals but increased UV exposure can lead to the disease in all ages, and the use of indoor tanning beds has added to the risk in younger population [2–5]. The majority of expenses associated with melanoma are for treating advanced stages of the disease, and early detection can save costs [6]. Survival rates for melanoma are lower compared to other types of cancers, making it particularly deadly [4]. Current methods for early detection of melanoma include physical exams based on the ABCDE criteria (asymmetry, border irregularity, color variegation, diameter, and evolving features) and technology-based solutions such as dermoscopy, image analysis, ultrasound, confocal microscopy, and optical coherence tomography [1, 6, 7]. Biopsies and various imaging techniques such as computed tomography and magnetic resonance imaging are also used to diagnose malignant melanomas [8, 9]. Blood tests, such as measuring lactate dehydrogenase (LDH) levels, can be used to check for the spread of the cancer in more advanced cases [9, 10]. Additionally, newer techniques like melanin absorption spectroscopy use the optical properties of melanin to diagnose malignant melanomas [7]. While these methods have improved diagnostic accuracy, a "good clinical eye" and the expertise of a pathologist are still necessary for accurate diagnosis [1]. There is also a high rate of disagreement among pathologists when it comes to diagnosing melanoma and benign melanocytic nevi. Improving diagnostic accuracy is important to decrease false positives and false negatives, which can have negative impacts on patients’ health and treatment [11]. Despite research on the active role of sensory and sympathetic nerves in melanoma growth and spread [12–15], there is currently no diagnostic tool that uses the bioelectric behavior of these nerves to detect melanoma in its early stages.

The autonomic nervous system (ANS) is a branch of the peripheral nervous system with two of its major branches playing antagonistic roles. The parasympathetic nervous system (PSNS) and sympathetic nervous system (SNS) are the branches with physiological—‘rest and digest’ and ‘fight and flight’ responses, respectively. Together, these nerve fibers control and regulate metabolism and homeostasis of the visceral organs by involuntary mechanisms. In human patients with melanoma, administration of β-blockers extended their survival [16]. β-adrenergic receptors are the primary effectors of the sympathetic nerve derived neurotransmitters, indicating a possible detrimental role of sympathetic nerves in human melanomas. Also, mice with denervated SNS nerves by sympathectomy procedure survived longer and developed larger melanomas [14]. On the contrary, both the genetic and chemical denervation of Nav 1.8+ sensory nerves accelerated the tumor growth in mice indicating protective role of Nav 1.8+ sensory nerves in murine melanomas and similar results were reported in human melanoma tumors [12]. Despite the antagonistic roles of sympathetic and sensory nerves in melanomas, there is no report of direct electrical recordings from melanomas. Also, it is not known if the tumoral nerves are electrically active and in which temporal pattern they display the electrical activity. Also, multiple population studies report that there is a significant difference in the occurrence, time course, and pattern of metastasis between men and women throughout the development of cutaneous melanomas [16, 17].

We hypothesize that innervated primary subcutaneous B16 metastasizing melanomas are characterized by detectable neural activity while their counterparts bearing low metastatic potential have significantly reduced neural activity. Neural growth has an active role in promoting neoplastic transformation from healthy skin to malignant primary tumor and this principle can be used as a diagnostic tool for early malignancy detection. Also, the sex specific hormonal implications of gonadocorticoides on melanoma activity can be explored by comparing the quantified electrical activity between male and female mice groups. Thus, understanding the temporal patterns of electrical activity during in-vivo progression of metastasizing and low metastatic potential bearing melanomas would illuminate the neurobiology of the tumors in accordance with the tumor’s potential for metastasis as well as sex-specific disease implications.

## 2 Materials & methods

All animal studies were approved by the Institutional Animal Center and Use Committee (IACUC) at Case Western Reserve University. Animal welfare took priority over experimental studies when it came to euthanasia or other interventions.

### 2.1 Murine tumor models

Melanoma cancer was developed using cell line B16-F10 (ATCC CRL-6475-LUC2; RRID: CVCL_A4CJ) and B16-F1 (ATCC CRL-6323-LUC2) which was obtained from American Type Culture Collection (ATCC) (Manassas, VA, USA). Both the murine melanoma cells express luciferase in order to permit bioluminescent imaging (BLI). The B16 cells were cultured in Dulbecco’s Modified Eagle’s Medium (DMEM) (ATCC 30–2002) containing 10% Fetal Bovine Serum (FBS) (ATCC 30–2020) and 1% Penicillin-Streptomycin Solution (ATCC 30–2300). The cells were maintained under low passage (less than 15) and were kept in a humidified atmosphere of 5% CO_**2**_ in air at 37°C. B16-F10 and B16-F1 inoculations were performed under administration of 2% isoflurane inhalant in 8 weeks old, 20 grams weighing C57BL/6J mice (Jackson Laboratories; RRID: IMSR_JAX:000664) on the dorsal left side. B16-F10 cell line, a highly metastatic melanoma cell line, where the cancer cells frequently metastasize to the cranial area, by crossing the blood-brain barrier. The B16-F10 cells which come in close contact with brain endothelial cells disrupted the tight and adherens junctions of cerebral endothelial cells and used (at least partially) the paracellular transmigration pathway [18]. Subcutaneous injections were performed with 5x10^**5**^, B16-F10 and B16-F1 cells suspended in DMEM. Equal number of male and female mice were used in each of the experimental groups, all provided with Alfalfa free diet and water was provided ad-libitum.

All the animal procedures were performed under 2% isoflurane anesthesia vapors in 1 L/min oxygen setting. In order to alleviate the sufferings, post invasive animal procedures pain medications were provided, immediately as well as for two consecutive days post any invasive procedures. For euthanasia purposes, under 2% isoflurane anesthesia in 1 L/min oxygen, anesthetized mice were perfused using 20 mL of 1X Phosphate Buffered Saline (PBS) solution followed by 20 mL of 4% Paraformaldehyde (PFA) solution injected via the heart. Under anesthesia, cervical dislocation of vertebral column was also performed as an additional measure of euthanasia in order to confirm the success of euthanasia procedure.

### 2.2 Chemical sympathectomy

Chemical sympathectomy solution was prepared by dissolving 6-hydroxydopamine (6-OHDA, 100 mg/kg body weight) in sterile saline along with 0.01% of ascorbate as a stabilizer. On the fourth and second day before cancer cell inoculation, the drug was injected intraperitoneally (IP). After these two initial injections, the drug was administered IP every five days to maintain the sustained effect of denervation.

### 2.3 Microneurography and neural recordings

Neural recordings were performed for thirty minutes for each day under 2% isoflurane in 1 L/min oxygen, for two weeks starting from day 6 post inoculation of cancer cells. Microneurography needles were inserted into the primary tumor mass. The microneurography instrumental setup is shown in S1 Fig in S1 File. By using a micromanipulator, the needles were inserted at the same depth (5 mm) and same distance apart (4 mm), daily, in order to record from the same location. The two recording needles were fixed to a holder and were maintained at a 4 mm separation distance. The holder was mounted on a 1μm, x-y-z resolution micromanipulator system, so that the daily insertion depth could be repeated with certainty. The neural data were sampled at 20 kHz and filtered from 500 Hz to 1200 Hz using a 7th order, zero-phase, digital bandpass filter. ADInstruments amplifier (FE285) was used for all the recordings. Recorded neural data were displayed and stored in Lab Chart files. Lab Chart files were exported into MATLAB (RRID:SCR_001622) compatible files and the results were quantified in terms of number of spikes per file using UltraMegaSort2000 (UMS2000) spike sorting program [19] and neural spikes were plotted for ten minutes. The averaged spike count per 10 minutes, was plotted as a function of days post inoculation of cancer cells.

### 2.4 Bioluminescence imaging

Tumor growth and metastasis in-vivo were tracked using bioluminescence imaging (BLI). Images were acquired using Perkin-Elmer’s In-Vivo Imaging System (IVIS) at 60s exposure. 200 μL of D-luciferin solution (12.5 mg/ml of D-luciferin in sterile phosphate buffer solution) was injected IP as a substrate ten minutes before acquiring the images. Total flux was quantified from the primary tumor region in order to quantify the in-vivo growth, as well as from the cranial region to quantify the growth of secondary metastatic foci. Same sized regions of interest (ROIs) were used for the images with the same Field of View (FOVs) for a particular day of imaging.

In order to find the specific day of metastasis, we statistically compared the bioluminescence flux from the baseline day (i.e. second day post cancer cell inoculation) with every consecutive day after that. The ‘common threshold day’ was computed when we observed significant difference from the baseline day to specific consecutive day. This calculation was performed individually for each of the experimental groups where all the animals from that particular experimental group were pooled for computing the common day on which we see significant difference from the baseline day. The average flux from that particular day, was used as a ‘threshold’, on which we observed significant statistical increment from the flux values on baseline day. Further, for each animal, the ‘individual day of metastasis’ was assigned for that particular day, from which we observed continuous increment above the calculated average ‘threshold’ flux value. Finally, the results pertaining to the ’individual day of metastasis’ were aggregated from the respective mouse groups and used for statistical comparison.

### 2.5 Histology

To confirm the presence of nerve fibers in primary melanoma tumors, histology was performed. At day 10 post-inoculation, the mice were perfused, and the primary tumors were fixed using 20 mL of 1X Phosphate Buffered Saline (PBS) solution followed by 20 mL of 4% Paraformaldehyde (PFA) solution injected via the heart. The excised tumor tissues were transferred to paraformaldehyde for 24 to 48 hours and refrigerated at 4°C. After fixing in paraformaldehyde, the samples were immersed in a 15% sucrose solution with 1X PBS for 24 hours at 4°C. Then, they were moved to a 30% sucrose 1X PBS solution and kept at 4°C until processing. The samples were formalin-fixed and paraffin-embedded, and 5μm thick slices were subjected to immunohistochemistry chromogenic detection using neurofilament primary antibody (NF, RRID: Thermo Fisher 2F11; TH, RRID: Sigma-Aldrich AB152) to detect the presence of nerve fibers and autonomic nerves, respectively. To obtain a comprehensive pathological understanding of the tumor microenvironment, slices were obtained from various tumor areas, ranging from about 100 μm. The immunohistochemical (IHC) staining process was conducted according to the manufacturer’s instructions. Number of events per square millimeter of tissue slices were calculated in order to quantify and compare the staining metrics. In the immunohistochemistry (IHC) results section, ‘N’ represents the number of mice while ‘n’ represents the number of tissue slices.

### 2.6 Statistics

All the statistical analysis was performed in the MATLAB software. The figures, figure captions as well as text in the results section, associated with each study provide information about the sample size, tests and significance level. The data is presented as mean values with standard deviations (mean ± S.D.). To determine the suitable statistical test, all datasets underwent a normalcy assessment using the ks-test. In cases where the data showed a normal distribution with two means, one-sample t-tests and paired t-tests were employed accordingly. For datasets showing non-normal distributions with two means, the rank-sum test and sign-rank test were utilized.

## 3 Results

### 3.1 Melanoma exhibit bioelectric activity

**Fig 1:**
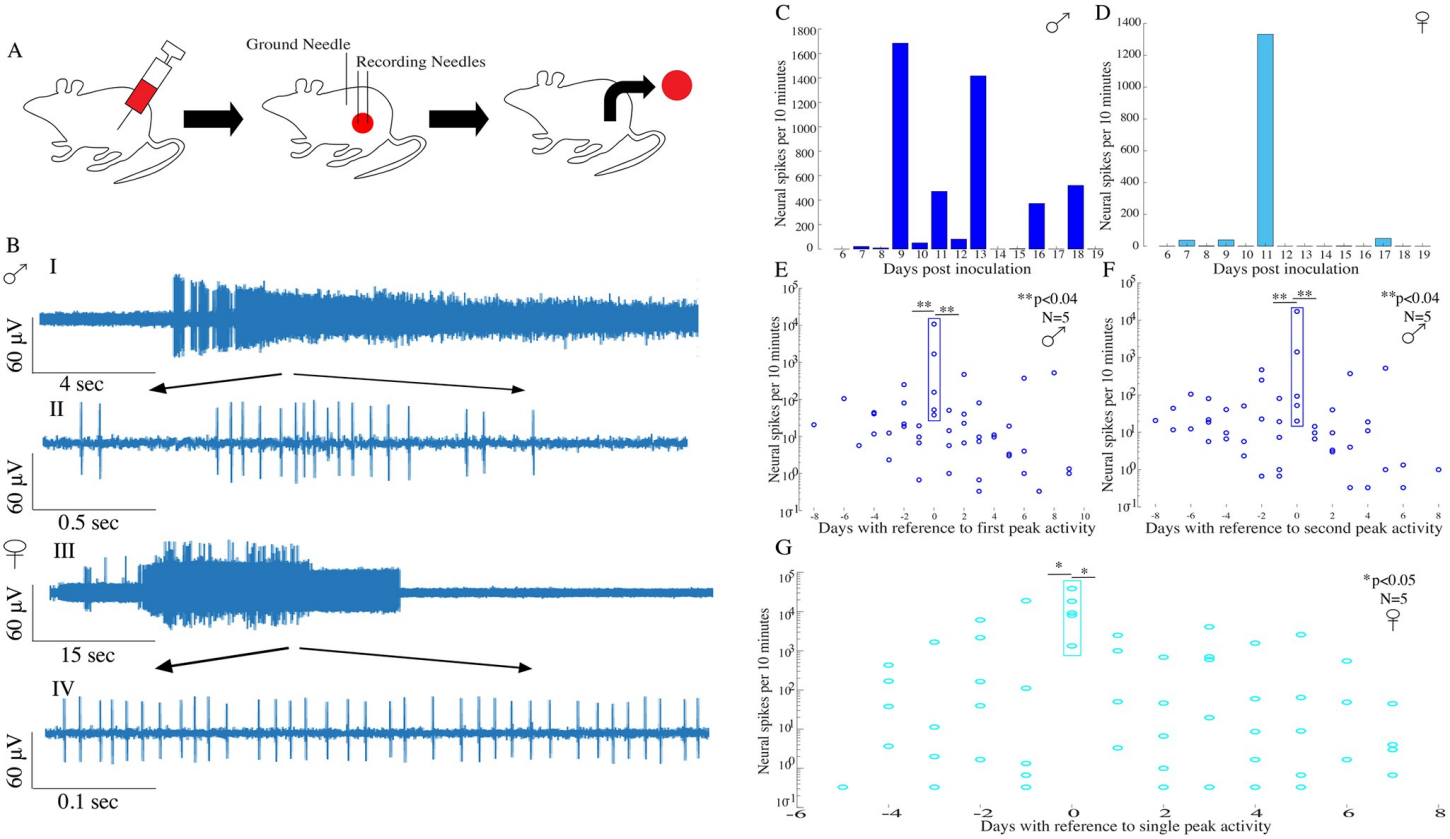
Bioelectric activity in B16-F10 melanoma tumors. **(A)** Mice were inoculated with half a million cancer cells. Post optimum tumor growth daily neural recordings were started and electrode positions are shown in the figure. Terminally, the tumors were harvested for immunohistochemical studies. **(B) (I)** Neural activity recorded from the metastasizing tumor in male mice. The magnified neural spikes are shown in **(II). (III)** Neural spiking activity recorded from the metastasizing tumor in female mice, while **(IV)** shows the magnified neural spikes in female mouse tumors. **(C)** An example of quantified neural spikes in a male mouse. **(D)** Daily neural spikes observed in a female mouse post inoculation of cancer cells. **(E)** A significant increase was observed in male mice during first peak activity recorded in melanoma tumors compared with a prior day of peak activity; while the next day the activity was significantly lesser compared with the peak day (N = 5; sign-rank test; p<0.04). **(F)** Also, significant increment was observed in male mice during second peak activity compared with a prior day of peak activity; while the next day the activity was significantly lesser compared with the peak day (N = 5; sign-rank test; p<0.04). **(G)** Similarly, in female mice there was significant increment during the peak activity compared with the prior and next day (N = 5; sign-rank test; p<0.05).

To explore in-vivo neural activity patterns in melanoma tumors, we inoculated the B16-F10 cells and waited for a period of 6 days for the tumor to reach a detectable size (~1.25x10^-7^ m^3^) and started daily neural recordings (Fig 1A). We observed significant neural spiking activity in the form of discontinuous trains in both male (Fig 1B. I) and female (Fig 1B. III) murine tumor activity. Fig 1B II and 1B IV show magnified neural spikes from male and female mice, respectively.

We then analyzed the patterns of neural activity during tumor progression, and plotted neural spike numbers observed each day as a function of days post-inoculation of cancer cells. The results show that neural activity is not continuously present, but the activity is observed to peak during specific days in both males and females. Fig 1C and 1D show the neural activity patterns from a male and a female mouse, respectively. In order to compare the occurrence of peak activity relative to the tumor growth, we referenced the peak day as day 0 and compared the neural spikes numbers on each peak day with the prior- and following- day of -peak activity. Since the peak activity in different mice, occurred on different days, we used the peak day/s as reference in order to visualize the data. In male mice, two peaks are considered separately. While generating the plot for first peak data, we considered the first peak day as day 0 and plotted the data with respect to that, excluding second peak data and vice a versa, for the second peak data. Without this renormalization, it would be difficult and confusing to visualize the peak activity as the peaks occur on different days in different mice. Fig 1E and 1F illustrate the individual data points showing neural spiking activity from 5-male and female mice, respectively. Further, Fig 1E shows that the first peak activity is significantly higher compared with a prior day (N = 5; sign-rank test; p<0.04) and next day (N = 5; sign-rank test; p<0.04). Similarly, the second peak activity is also significantly higher compared with a prior day (N = 5; sign-rank test; p<0.04) and next day (N = 5; sign-rank test; p<0.04) as shown in Fig 1F. However, in female mice only a single day with high spiking activity was observed and the peak occurred on different days in different mice. Thus, similarly using peak day as a reference, we plotted the daily neural spikes as a function of days post inoculation as shown in Fig 1G. The peak spiking activity was observed to be significantly higher when compared with the prior (N = 5; sign-rank test; p<0.05) and following day (N = 5; sign-rank test; p<0.05) as shown in Fig 1G. These results indicate that melanoma tumors are electrically active with high peak activities similar to breast tumors [20].

### 3.2 Male mice display two peaks of activity while female mice display a single peak of activity

**Fig 2:**
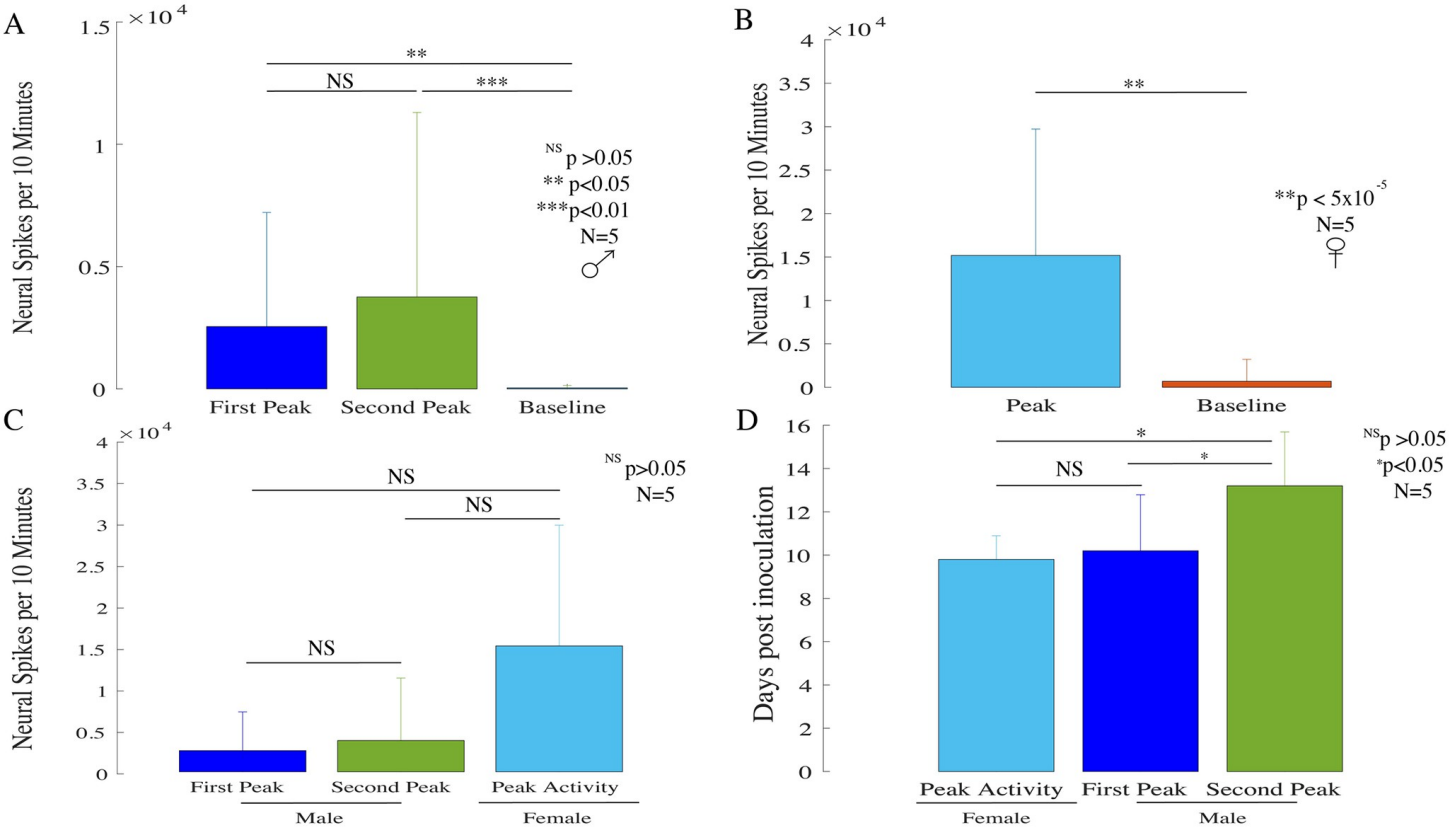
Sex-specific patterns in neural activity within B16-F10 tumors. **(A)** In male mice, neural spikes observed in first and second peak activity were significantly higher compared with the average neural spikes in neurally non-active days i.e. baseline. (first peak: N = 5; p<0.05; rank-sum test; second peak: (N = 5; p<0.01; rank-sum test) **(B)** Neural spikes observed in female peak activity were significantly higher compared with the average neural spikes found in baseline activity. (N = 5; p<5x10^-5^; rank-sum test) **(C)** Also, neural spikes in both the male peaks and female peak activity are not different (N = 5; p>0.05; two-sample t-test). **(D)** First peak in the male mice occurred at the same time as the single female peak activity (N = 5; p>0.05; rank-sum test), while second peak in the male mice occurred later than male first peak (N = 5; p<0.01; signed-rank test) and female peak activity (N = 5; p<0.05; rank-sum test).

To explore further the difference between male and female neural firing in tumors as the cancer progresses in vivo, we compared the average neural spikes from peak activity of each group. The two peaks observed in the neural activity in male mice, are termed as ‘first peak’ and ‘second peak’. Fig 2A shows that the average neural spikes numbers in the first (N = 5; p<0.05; rank-sum test) and second peaks (N = 5; p<0.01; rank-sum test) are significantly higher compared to the average spikes found in the neurally non-active days i.e. baseline. Also, no difference was observed between the neural spike counts in the first and second peak (N = 5; p>0.05; paired t-test). Further, Fig 2B shows that average neural spikes in single female peak activity are significantly higher compared to the average baseline neural spike activity (N = 5; p<5x10^-5^; rank-sum test). No difference was noted in the average neural spikes found between the two neural activity peaks in males and the single female neural activity peak as shown in Fig 2C (N = 5; p>0.05; two-sample t-test). Also, the first peak in the male mice occurred at the same time as that of the female peak (Day 10; N = 5; p>0.05; rank-sum test) while the second peak in the male mice appeared significantly later than the first peak in the male mice (N = 5; p<0.01; signed-rank test) and the peak day in female mice (N = 5; p<0.05; rank-sum test) as shown in Fig 2D. Thus, these results strongly indicate that there is a sex-specific pattern in the recorded neural peak activity in melanoma rodent tumors.

### 3.3 Melanomas with low metastatic potential, have significantly lower neural activity

To determine if metastasizing and low metastatic potential bearing tumors display different patterns of neural activity, we performed daily neural recordings from the low metastatic potential bearing tumors obtained from B16-F1 cells. Neural data were recorded from the B16-F1 tumors and processed using the similar methods used for the data obtained from metastasizing tumors.

**Fig 3:**
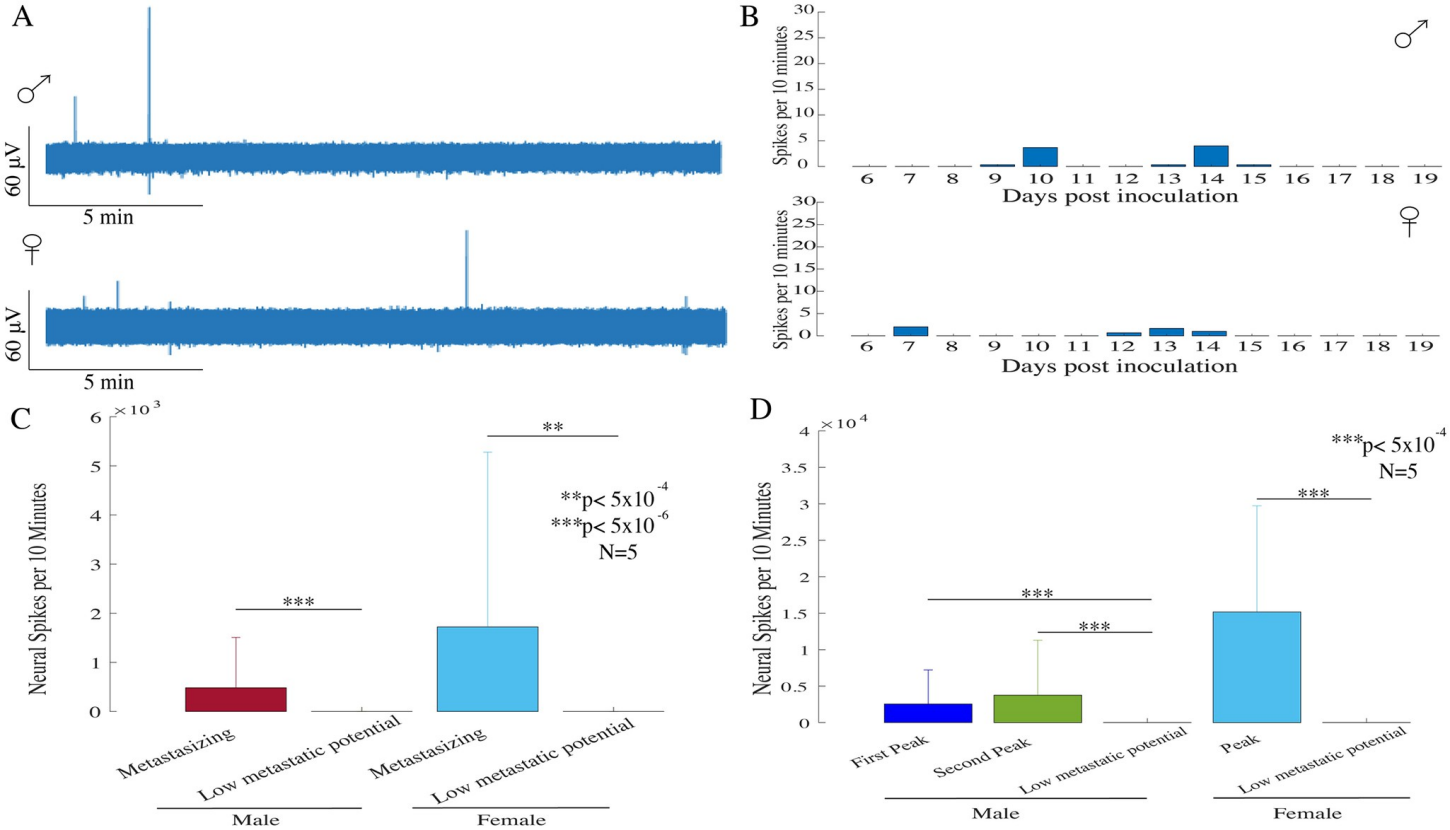
Neural recordings in B16-F1 low metastatic potential bearing tumors. **(A)** Example of neural activity recorded from a male and female mouse bearing tumor with low metastatic potential. **(B)** Daily neural activity pattern from a male and a female mouse bearing tumor with low metastatic potential. (**C)** Both the male (N = 5; p<5x10^-6^; rank-sum test) and female (N = 5; p<5x10^-4^; rank-sum test) mice, showed significantly reduced average neural spiking activity in mice bearing tumors with low metastatic potential, compared with the average neural activity observed in metastasizing tumor bearing mice in pertinent groups. **(D)** In male mice bearing tumors with low metastatic potential, significantly reduced average neural activity was reported when compared with the first and second peak activity (N = 5; p<5x10^-4^; rank-sum test). Also, in female mice bearing tumors with low metastatic potential, significantly reduced average neural activity was observed compared with the female peak activity (N = 5; p<5x10^-4^; rank-sum test).

Fig 3A shows the ENG recorded from a single male and a female mouse bearing B16-F1 derived low metastatic potential bearing tumor. During a 30-minute period of ENG activity, there was a significant reduction in neural spiking activity observed in recordings from both mice. Fig 3B shows the neural activity pattern from a male and a female mouse bearing tumor with low metastatic potential. The average number of neural spikes were significantly reduced in the low metastatic potential bearing male (N = 5; p<5x10^-6^; rank-sum test) and female (N = 5; p<5x10^-4^; rank-sum test) mice compared to their respective metastasizing counterparts, as shown in Fig 3C. Also, significant reduction in the average neural spikes in non-metastasizing tumors was reported compared to the peak neural activity spikes in each sexual group (N = 5; p<5x10^-4^; rank-sum test), as demonstrated in Fig 3D. Thus, our results indicate that tumors with low metastatic potential, lack the neural peak activity indicating the high neuroelectric activity is a unique feature of metastasizing tumors.

### 3.4 Sympathectomy suppresses neural activity in males and females

The sympathetic nervous system (SNS) has several important functions in the regulation of multifarious factors in tumor neural microenvironment such as immune response, angiogenesis, mesenchymal activation, oncogene activation. We therefore tested the hypothesis that the neural activity recorded within metastasizing melanomas originates from the peripheral sympathetic nerves. We then performed a chemical sympathectomy by intraperitoneal administration of 6-OHDA neurotoxins and recorded the neural activity within the tumor. The neurotoxin dose administrations were started before the cancer cell inoculation and frequently repeated in order to maintain the continuous effect of denervation as described in the methods section.

**Fig 4:**
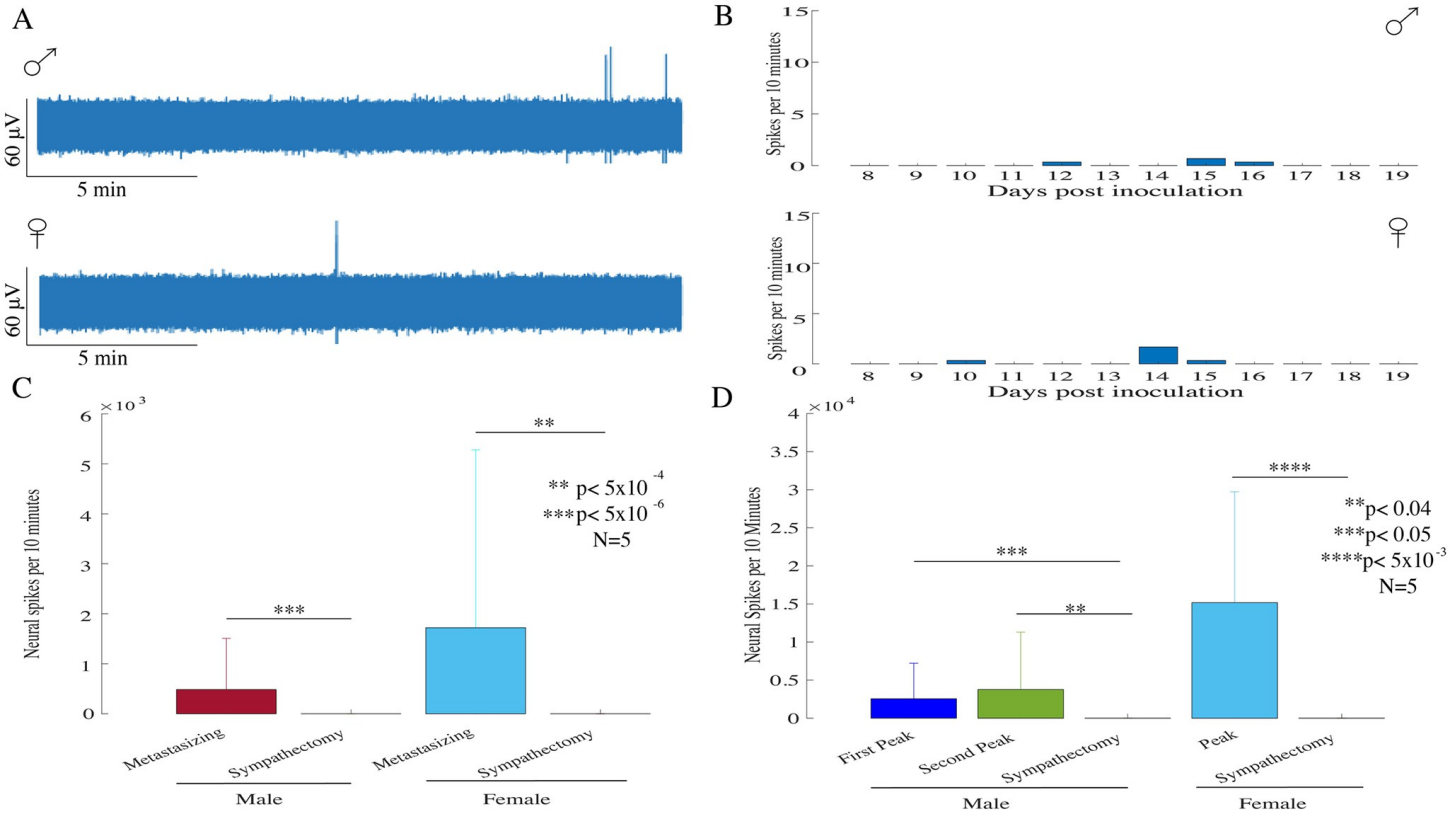
Neural activity recorded in B16-F10, sympathectomized tumor bearing mice. **(A)** ENG signals recorded from a sympathectomized male as well as female mouse bearing B16-F10 derived melanomas. **(B)** Daily neural activity pattern from a male- and a female- sympathectomized mouse bearing metastasizing tumors. **(C)** Both the male (N = 5; rank-sum test; p<5x10^-6^) and female (N = 5; rank-sum test; p<5x10^-4^) mice, showed significantly reduced average neural spiking activity in sympathectomized mice compared with the average neural activity observed in untreated metastasizing tumor bearing mice. **(D)** In sympathectomized male mice, significantly reduced average neural activity was reported when compared with the first and second peak activity (first peak: N = 5; two sample t-test; p<0.04 & second peak: N = 5; two sample t-test; p<0.05). Also, in sympathectomized female mice, significantly reduced average neural activity was observed compared with the female peak activity (N = 5; two sample t-test; p<5x10^-3^).

Fig 4A shows examples of neural activity obtained from a sympathectomized male and female mouse bearing B16-F10 metastasizing melanoma. The 30-minute window of recording of neural activity shows very little activity in the two examples. The neural activity is then plotted over the course of 11 days (Fig 4B) and the results show that the neural activity has been eliminated over the entire recording period for both sympathectomized male and female mice. We then compared the neural spikes counts from metastasizing and sympathectomized tumors in male and female groups, and observed a significant reduction in average spiking activity in male (N = 5; rank-sum test; p<5x10^-6^) and female (N = 5; rank-sum test; p<5x10^-4^) mice described in Fig 4C. Average neural activity was also significantly lower compared with the average peak activity in male (first peak: N = 5; two sample t-test; p<0.04 & second peak: N = 5; two sample t-test; p<0.05) as well as female (N = 5; two sample t-test; p<5x10^-3^) mice as shown in Fig 4D. Thus, our results show that sympathectomy completely suppresses the peak activity in both the groups indicating that the origin of this activity lies in tumoral sympathetic nerves.

### 3.5 Secondary distant metastasis has sex-specific implications and correlates with the neural activity

Previous studies have reported the direct role of SNS in initiating secondary distant metastasis and survival [14, 21, 22]. To examine the implications of specific sex-associated factors as well as denervation of the sympathetic nervous system in secondary distant metastasis, we computed the day on which metastasis to the cranial area was noted which a common site of distant metastasis in melanomas [23] by measuring the daily bioluminescence flux. The details about, how ‘the day of metastasis’ was detected, are included in methods section.

**Fig 5:**
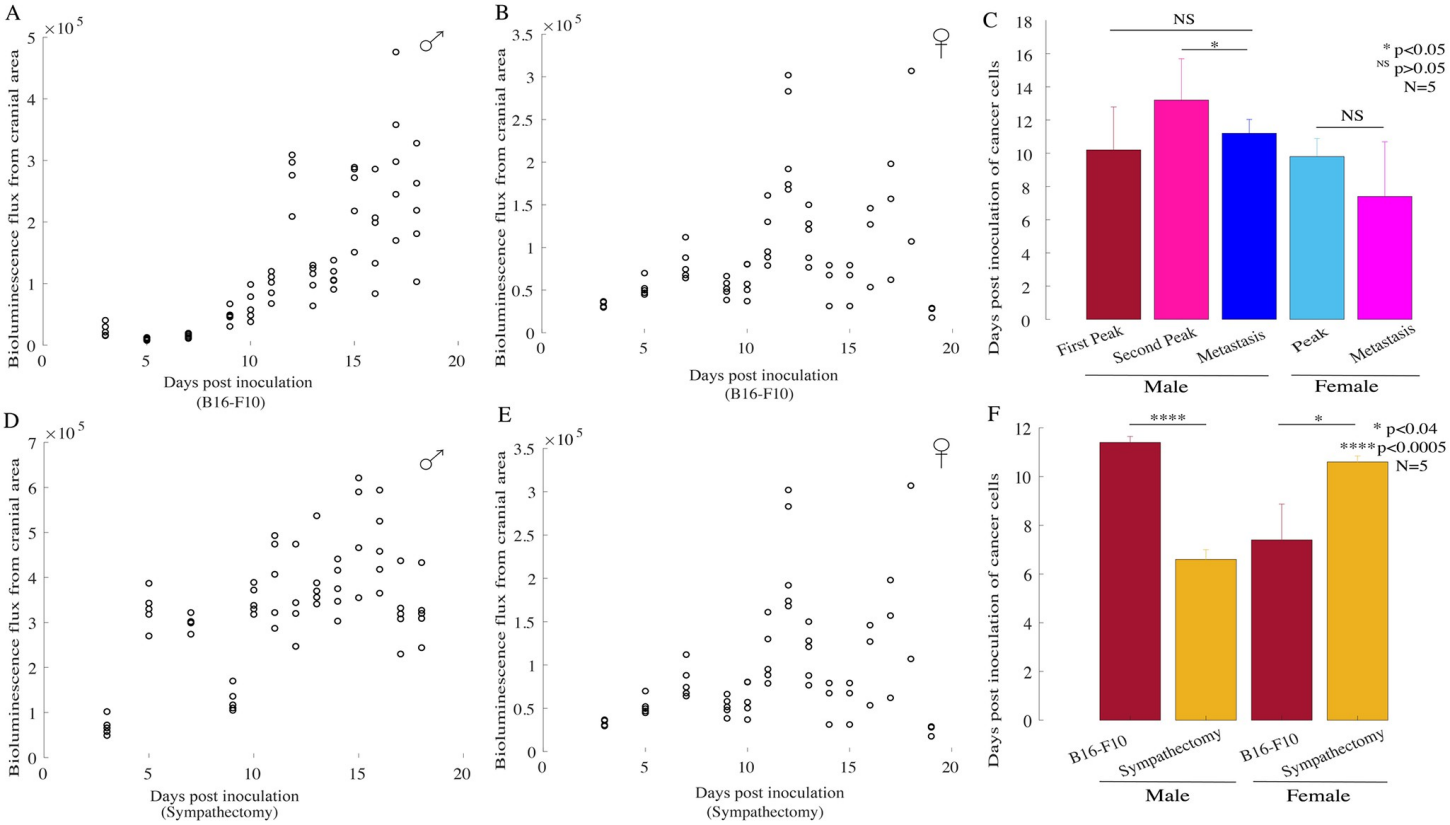
Implications of sex-specific factors and sympathectomy on secondary distant metastasis with B16-F10 tumors. Bioluminescent signal flux is shown from the B16-F10 tumor bearing mice from **(A**) male subjects and **(B)** female subjects **(C)** Neural peak activity was correlated with the day of distant metastasis to cranial areas in both the sexual groups. In male mice, the first peak in neural activity occurred at the same time as the secondary distant metastasis (N = 5; two sample t-test; p>0.05), while the second peak occurred later (N = 5; two sample t-test; p<0.05). In female mice as well, the peak activity was observed at the same time as the secondary distant metastasis (N = 5; two sample t-test; p>0.05). Bioluminescent signal flux is shown from the tumor bearing sympathectomized mice from **(D)** male subjects **(E)** female subjects **(F)** Sex specific implications were observed on the detected distant metastasis to the cranial area where metastasis was observed significantly earlier in male mice (N = 5; two-sample t-test; p<0.0005) while it was delayed in female mice (N = 5; two-sample t-test; p<0.04).

Bioluminescence flux signal distribution from the cranial area during the in-vivo cancer progression in male and female mice bearing B16-F10 melanomas is shown in Fig 5A & 5B, respectively. Bioluminescent images from the male and female mice are shown in S2 Fig in S1 File. To explore the temporal correlation between the occurrence of neural peak activities observed in the B16-F10 derived tumor bearing mice and the secondary distant metastasis days, we compared the peak activity days of the particular sex group, with the days on which distant metastasis to cranial area was observed in the pertinent group. As shown in Fig 5C, our results show that in male mice the first peak activity occurred simultaneously with the secondary distant metastasis (N = 5; two sample t-test; p>0.05) as there is no difference reported, while single peak activity in female mice was also correlated with the secondary distant metastasis (N = 5; two sample t-test; p>0.05). The second peak activity in male mice occurred significantly later than the day of metastasis (N = 5; two sample t-test; p<0.05). Also, temporal correlation between neural peak activities and the distant metastasis, suggests that neural activity in the tumor microenvironment plays a crucial role in tumor fate, in both sexes.

We also analyzed the sex differences of bioluminescence flux signal distribution from the cranial area during the in-vivo cancer progression in male (Fig 5D) and female (Fig 5E) sympathectomized mice bearing B16-F10 melanomas. Bioluminescent images from sympathectomized male and female mice are shown in Supplementary section S3 Fig in S1 File. Sympathectomy caused significantly earlier metastasis in male mice (N = 5; two sample t-test; p<0.0005) while metastasis occurred significantly later in female mice (N = 5; two sample t-test; p<0.04) undergone sympathectomy, as shown in Fig 5F. Thus, these results indicate that sympathectomy exerted a contrasting effect on the detected distant metastasis to cranial area.

It is well known that B16-F1 derived tumors have low metastatic potential compared to B16-F10 derived highly metastasizing tumors. In order to validate the growth characteristics and metastatic potential of the two tumor models, we compared the average bioluminescence flux computed from primary tumor region as well as cranial region. We observed significantly reduced average bioluminescence flux in low metastatic potential tumor bearing mice in primary tumor mass area in both the sexes i.e. in female mice (N = 5, rank-sum test, p<5x10^-4^) as well as male mice (N = 5, rank-sum test, p<0.05). These results are shown in S4A & S4B Fig in S1 File. Similar results were observed with significantly reduced average bioluminescence flux in low metastatic potential tumor bearing mice, in cranial area in both the sexes i.e. in female mice (N = 5, rank-sum test, p<5x10^-3^, S4C Fig in S1 File) as well as in male mice (N = 5, rank-sum test, p<0.05, S4D Fig in S1 File). These results from comparison among the average flux in primary tumor mass region in both the sexes implicate that B16-F1 derived low metastatic potential bearing primary tumors grow at slower pace compared to B16-F10 derived highly metastasizing tumors. Also, the similar results observed from comparison among the average flux in cranial region in both the sexes validate the low metastatic nature of B16-F1 tumors, as significantly reduced average flux was noted in cranial area; a prevalent site for distant metastasis in melanomas, in the B16-F1 derived low metastatic potential bearing tumors compared to B16-F10 derived highly metastasizing tumors.

Also, to explore the effect of the sympathectomy procedure on the primary tumor mass, we compared the average bioluminescence flux observed from primary tumor mass region in B16-F10 metastasizing tumor bearing mice with sympathectomized mice with B16-F10 metastasizing tumors. Comparison among bioluminescence flux from primary tumor mass region in B16-F10 metastasizing tumor bearing mice and sympathectomized mice with B16-F10 metastasizing tumors is shown in S5 Fig in S1 File, where in female mice (N = 5; rank-sum test, p<5x10^-6^) as well as males (N = 5, rank-sum test, p<5x10^-3^) sympathectomy caused the reduction in the average bioluminescence flux quantified from the primary tumor masses, indicating the beneficial protective role of sympathectomy procedure on the primary tumor masses in both the sexes.

### 3.6 Immunohistochemical analysis shows that nerve density is higher in metastasizing tumors compared to tumors with low metastatic potential

In order to quantify the difference in nerve density within metastasizing- and low metastatic potential bearing- primary tumor tissues, we performed immunohistochemical analysis to detect the presence of nerve fibers as well as sympathetic fibers using neurofilament (NF) and tyrosine hydroxylase (TH) antibodies.

**Fig 6:**
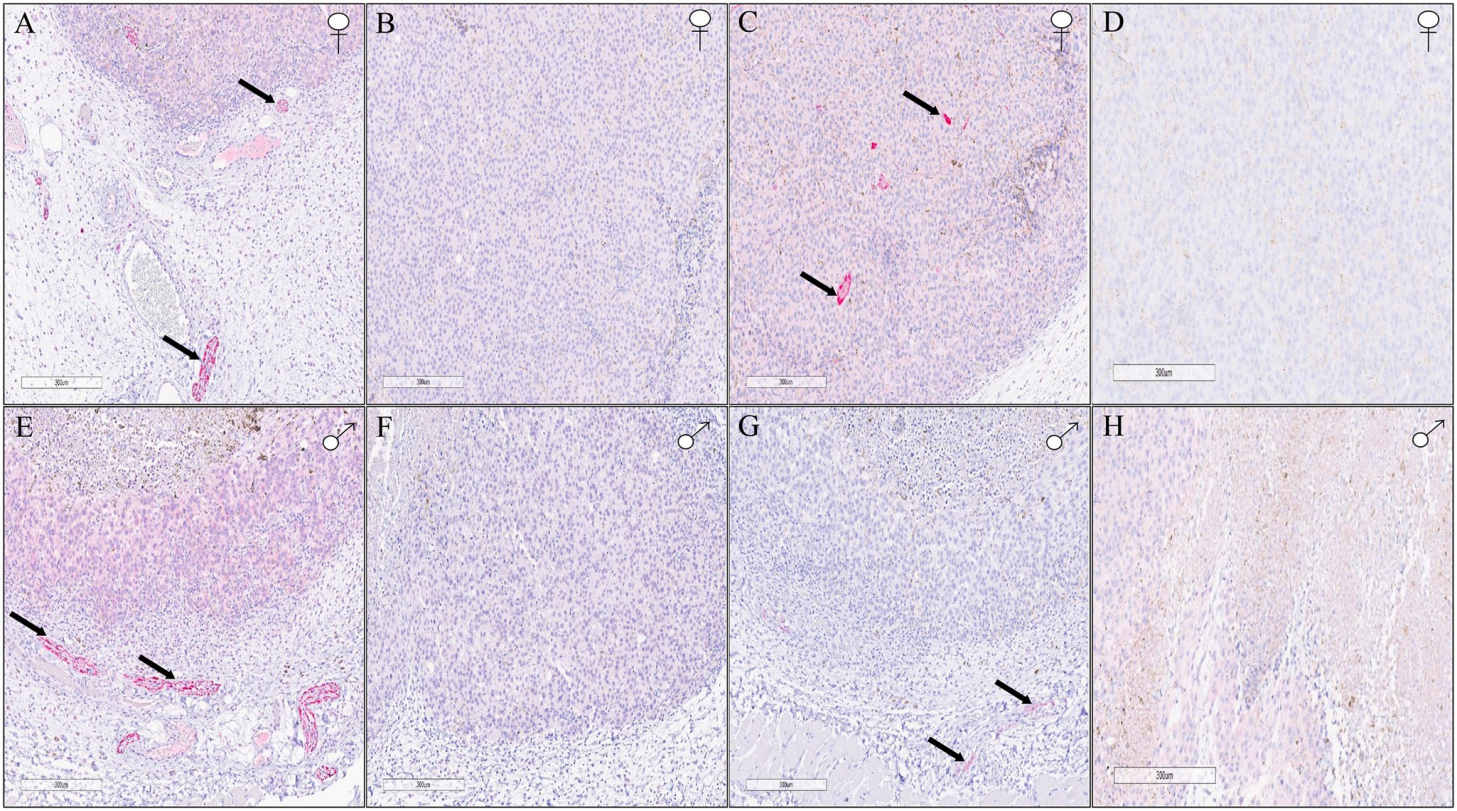
Illustration of neural staining in B16-F10 derived highly metastasizing melanoma tumors and melanoma tumors with low metastatic potential obtained from B16-F1 cell line. Exemplary images of melanoma primary tumor tissue slices stained with NF stain, from female mice are shown in panels (A) and (B) where panel **(A)** shows an image from metastasizing melanoma tumor slice while panel **(B)** shows an image from low metastatic tumor slice. Also, exemplary images of melanoma primary tumor tissue slices stained with TH stain, from female mice are shown in panels (C) and (D) where panel **(C)** shows an image from B16-F10 derived melanoma tumor slice while panel **(D)** shows an image from B16-F1 derived tumor slice. Similarly, exemplary images of primary tissue slice from male mice stained with NF stain, are shown in panels (E) and (F) where panel **(E)** shows an image from B16F-10 derived melanoma tumor while panel **(F)** shows an image from B16-F1 derived tumor. Also, exemplary images of primary tissue slice from male mice stained with TH stain, are shown in panels (G) and (H) where panel **(G)** shows an image from metastasizing melanoma tumor while panel **(H)** shows an image from low metastatic potential bearing tumor.

We show exemplary images from NF-stained tissue slices from female- metastasizing and low metastatic potential bearing -tumor bearing mice in Fig 6A and 6B, respectively. Also, Fig 6C and 6D show the images of tissue slices from primary melanomas stained with TH from female mice bearing metastasizing and low metastatic potential bearing -tumors, respectively. Similarly, images from NF stained tissue slices from male- metastasizing and low metastatic potential bearing, tumor bearing mice are shown in Fig 6E and 6F, respectively. Further, Fig 6G and 6H show the images of primary melanomas stained with TH from male mice bearing metastasizing and low metastatic potential bearing -tumors, respectively.

**Fig 7:**
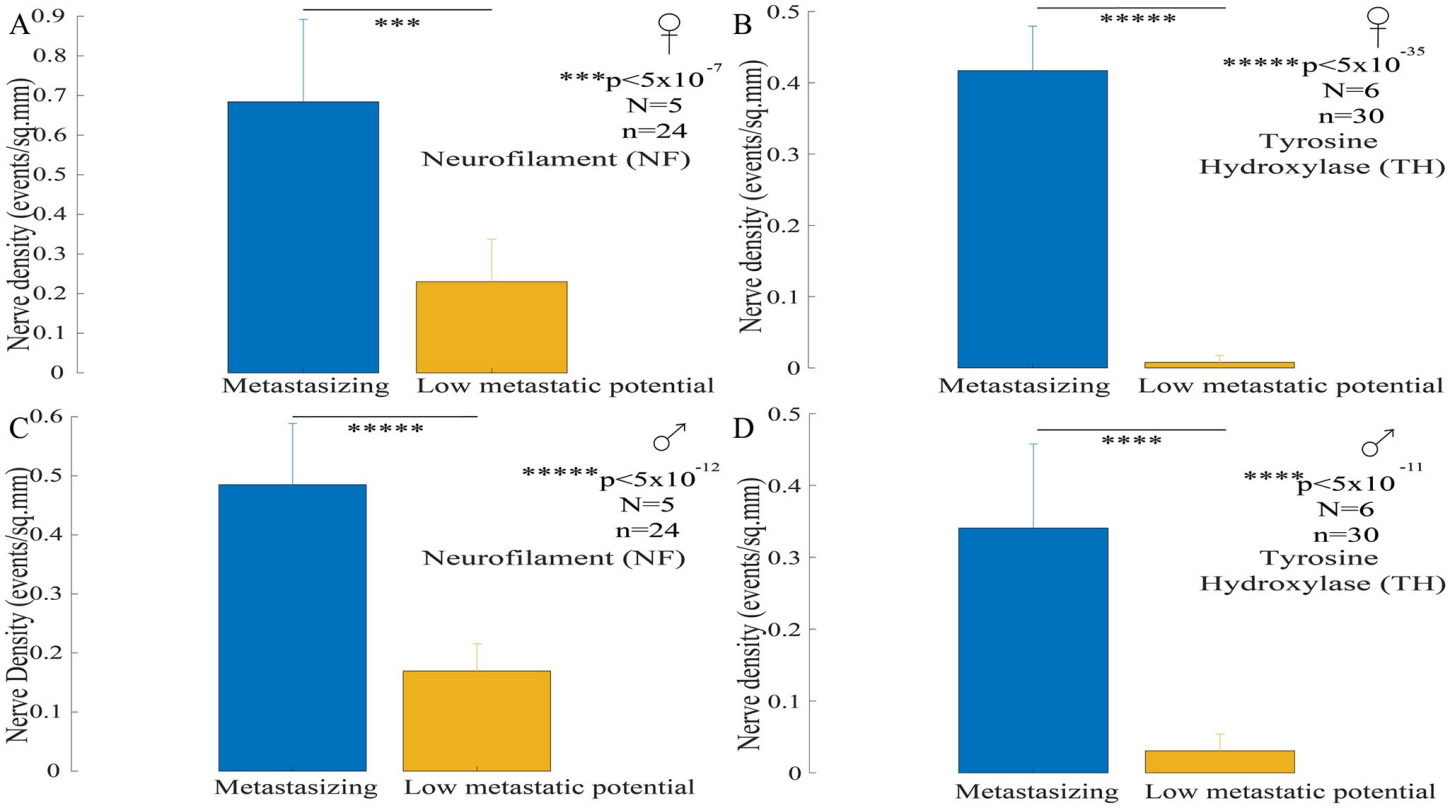
Comparison of neural density in metastasizing and melanoma tumors with low metastatic potential. **(A)** Significantly reduced nerve density is observed in melanomas with low metastatic potential compared to metastasizing tumors (N = 5; n = 24; two-sample t-test; p<5x10^-7^) in female mice. **(B)** Metastasizing melanomas were found with significant higher density of sympathetic nerve fibers compared to melanomas with low metastatic potential in female tumor bearing mice (N = 6, n = 30, two-sample t-test, p<5x10^-35^). **(C)** Similar observation is found in male mice as well; where significantly reduced nerve density is observed in melanomas with low metastatic potential compared to metastasizing tumors (N = 5; n = 24; two-sample t-test; p<5x10^-12^). **(D)** Also, similar results were observed in male mice for TH staining as well, where metastasizing melanomas were found with significant higher density of sympathetic nerve fibers compared to melanomas with low metastatic potential (N = 6; n = 30, two-sample t-test, p<5x10^-11^).

Further, quantitative analysis for NF stain, comparing nerve density among the female- metastasizing and low metastatic potential bearing -tumor bearing mice is shown in Fig 7A. Significant higher nerve density is observed in metastasizing tumors compared to low metastatic potential bearing tumors (N = 5; n = 24; two-sample t-test; p<5x10^-7^). In female mice, metastasizing melanomas were also found with significant higher density of sympathetic fibers compared to melanomas with low metastatic potential as shown in Fig 7B. We noted similar results in male mice for both the stains, where significant higher nerve density is observed in metastasizing tumors compared to low metastatic potential bearing tumors for NF staining (N = 5; n = 24; two-sample t-test; p<5x10^-12^) as well as TH staining (N = 6; n = 30, two-sample t-test, p<5x10^-11^), shown in Fig 7C & 7D.

These results implicate that overall nerve density is correlated with the metastasizing potential of the tumors. Also, these results indicate that sympathetic innervation density, is correlated with the metastasizing potential of the tumors, since metastasizing melanomas showed significantly higher nerve density compared to their counterparts with low metastatic potential.

Further, we compared the nerve density among male and female mice bearing B16-F10 metastasizing melanoma tumors. We observed higher nerve density in female mice compared to their male counterparts in both the, NF (N = 5; n = 24, two-sample t-test, p<0.05) and TH (N = 6; n = 30, two-sample t-test, p<0.05) stained tumor slices, shown in S6 Fig in S1 File. Also, no difference was observed among the nerve density among male and female mice bearing B16-F1 non-metastasizing melanoma tumors for both the stains (NF: N = 5; n = 24, two-sample t-test, p>0.05; TH: N = 6; n = 30, two-sample t-test, p>0.05).

Thus, in summary, we observed elevated nerve density in spontaneously metastasizing tumors in both male and female mice, in contrast to tumors with low metastatic potential. These consistent findings were also mirrored in tumors subjected to sympathetic fiber staining (TH). Notably, we detected increased nerve density in tumors from female mice, encompassing both overall nerve fibers (NF) and sympathetic fibers (TH), compared to murine male tumors.

## 4 Discussion

Melanomas have nerve fibers [12, 13, 24], but there are no studies reporting direct electrical recordings from these tumors. The specific origin of the neuroelectric activity in melanomas is also unknown. The principle aim of this study was to record the neuroelectric activity of murine melanoma tumors in order to identify the unique pattern of spiking activity as tumors progress in vivo and its implications in sexes. The study also sought to explore the origin of the neural activity in the specific branch of the autonomic nervous system. The results of our study showed that melanoma tumors are electrically active and exhibit sex-specific temporal patterns. The neural activity in melanomas originates from the peripheral noradrenergic sympathetic nerves.

Multiple histological studies have confirmed the presence of nerves and nerve growth promoting factors in the skin during the early stages of neoplastic transformation, as well as in various clinical stages of primary subcutaneous melanomas. Sympathetic fibers were observed to be traveling with tumor vasculature and innervating tumor parenchyma [25, 26], and the number of nerve fibers decreased with increasing thickness of primary melanomas [12, 13]. Other studies also found strong evidence that Nav1.8+ sensory nerves and noradrenergic sympathetic nerves innervate B16-F10 cells obtained orthotopic melanomas in C57BL/6J mice [12, 14]. Depletion of sensory nerves in melanomas through chemical and genetic means resulted in accelerated growth and angiogenesis [12]. Additionally, Nav1.8+ sensory nerves play a preventative role in melanoma growth and angiogenesis, and their ablation via genetic and chemical means favors primary tumor progression [12]. Pharmacological ablation of sympathetic nerves through chemical sympathectomy significantly delayed tumor development, but the sympathetic nervous system favors metastasis in melanoma [14, 21, 26]. Interestingly, upregulation of genes associated with sensory nerves correlated with better outcomes particularly in human cancer patients [12]. Our study found that male C57BL/6J mice with B16-F10 derived metastasizing melanomas exhibited two peaks of high spiking activity, while their female counterparts only showed a single peak of activity during in vivo tumor growth. Also, these peak activities in different mice occur on different days post cancer cell inoculation, instead of the exact same day, both in male and female mice. Although, we do not know the exact reason behind peak activities being observed on different days, we hypothesize that the time specific changes associated with tumor immune microenvironment cause the peak activity to be time varying. Thus, we suggest that presence of neural activity in melanoma tumors with peakwise patterns indicate that these nerve fibers are not active continuously but at very specific times and this electrical signaling could be associated with specific physiological changes in the tumor microenvironment of melanomas. Further, we do not know why the intensity of neural spikes per ten minutes was higher in female mice, we hypothesize that since female mice showed only one peak as opposed to two peaks in male mice, the peak activity became more pronounced in female mice to obtain the similar effect in the tumor microenvironment.

Sensory and sympathetic nerves have been found in melanocytic nevi and primary subcutaneous melanomas. Nerve growth factor (NGF) is a factor that promotes neurite growth and differentiates sensory and sympathetic neurons [13]. Higher expression of NGF was detected in melanocytic nevi during the early stages of neoplastic transformation to primary melanomas [13]. There is an inverse correlation between an abundance of nerve fibers in NGF receptor (NGF-R) poor nevi and high expression of NGF-R in melanomas [13], indicating the potential role of NGF-R and nerve fibers in the neoplastic transformation of metastasizing melanomas. We observed that male and female mice bearing B16-F1 derived melanomas with low metastatic potential had no neural peak activity. This result indicates that neural activity is a key player in the malignancy initiation and metastatic transformation where tumors with low metastatic potential might altogether have reduced nerve innervation.

In C57BL/6J mice, the sympathetic nerves play a significant role in the development and growth of melanoma tumors, with physiological stress accelerating tumor growth through β-adrenergic receptors [14, 27]. The sympathetic nervous system (SNS) has regulatory roles in the tumor microenvironment, affecting inflammation, immune response, epithelial to mesenchymal transition (EMT), oncogene activation, angiogenesis, apoptosis, DNA damage repair, hematopoiesis, and other factors through β-adrenergic signaling [15]. Additionally, the SNS affects a variety of cancer-associated cells, including adipocytes, epithelial cells, fibroblasts, lymphoid and myeloid immune cells, vascular myocytes, pericytes, neural cells, and glial cells [28–30]. The signaling pathway is also critical in regulating the immune response in the tumor microenvironment, promoting macrophage recruitment and the transcription of IL-6 and IL-8 while suppressing the cytotoxic functions of T lymphocytes and NK cells [31–35]. The SNS has two pathways for enacting its physiological effect: innervation via the peripheral nerves throughout the body and a hormonal division that exerts the same effects as the noradrenergic nerves. Injecting 6-OHDA via IP injection does not affect central nervous system (CNS) neurons since the blood-brain barrier (BBB) does not allow the neurotoxin into the CNS [36, 37], suggesting that all recorded neural activity within melanomas comes from peripheral sympathetic nerves innervating the tumors, excluding the role of cranial sympathetic nerves. Furthermore, in ovarian carcinoma, it has been found that tumor-innervating local sympathetic nerves actively secrete adrenaline and noradrenaline into the tumor microenvironment, as the levels of these catecholamines were significantly higher in the TME than in the blood [38, 39]. We observed that injecting the neurotoxin 6-OHDA in C57BL/6J mice with B16-F10 metastasizing melanomas resulted in a reduced neural peak activity in both male and female groups. Perhaps, 6-OHDA was specifically chosen to investigate the branch specific role of peripheral sympathetic nerves in, in-vivo tumoral neural activity eliminating possibility that recorded signal is originated from the cancer cells, as post intraperitoneal injection 6-OHDA is actively and selectively transported to NE receptor to nerve termini, thereby destroying the nerve termini. Thus, we conclude that the neural activity recorded from melanomas originated in peripheral sympathetic nerve fibers. Further, the sympathectomy procedure was begun before inoculation of the cancer cells, in order to confirm the complete elimination of sympathetic activity from start of the experiment instead of later time point of experiment where there will be still some of the effect of sympathetic nerve activity. Perhaps, we think that injecting 6-OHDA before cancer cell inoculation could affect the tumor development in earlier duration of tumor growth (even in the time between inoculation of cancer cells and first day of neural recordings), causing the delay in metastasis we observed and this could not be achieved with the injection of neurotoxin in later time point during in-vivo progression of tumors.

When comparing the neural activity patterns between male and female mice with metastatic melanomas, we found that male mice had two peaks of neural activity while female mice had only one peak. Previous studies have shown that there are notable differences in the factors that affect disease progression and metastasis in males and females. In the United States, melanomas are more common in men than in women, and the location of the tumors also differs between the sexes [40]. Men tend to have melanomas in the trunk region while women more frequently have them on their lower limbs [17]. Although the total rate of metastasis is higher in men with cutaneous melanomas, women tend to have better survival outcomes after the development of distant metastasis [16]. Furthermore, women tend to have more locoregional metastasis than men, and their distant metastasis is observed later than in men [16]. These factors have contributed to better overall outcomes for women. Since, our results indicate that male mice showed two peaks of activity while female mice had only single peak, we can infer that higher neural activity may be associated with poorer disease outcomes by looking at the previous trends in disease progression. Also, sympathectomy exerted contrasting effects in both the sexes. Although, we do not know the specific reason behind contrasting effect exerted by sympathectomy in male and female mice, we hypothesize that the contrasting outcomes can be associated with the antagonistic hormonal implications in different sexes. Further, we also found higher nerve density in female mice, and this can be indicative of better outcomes associated with higher nerve density yet accompanied by lower activity.

In summary, the present results indicate that melanoma tumors exhibit electrical activity, with distinct patterns in male and female metastasizing tumor-bearing mice. Male mice display two distinct peaks of neural activity, while female mice exhibit a single peak. This neural activity originates from peripheral sympathetic nerve fibers, as sympathectomy completely eliminates the peaks in both male and female mice. Also, mice bearing tumors with low metastatic potential displayed significantly lowered neural spiking activity compared to their metastasizing tumor bearing counterparts. Moreover, the occurrence of peak neural activity is closely associated with the detection of distant metastasis to the cranial area. In male mice, the first peak coincides with the timing of detected metastasis, while in female mice, the single peak aligns with the detection of metastasis. Also, sympathectomy has contrasting effects on distant metastasis in male and female mice. It accelerates metastasis in male mice but delays it in female mice. Lastly, we observed a higher density of nerve fibers in spontaneously metastasizing tumors compared to tumors with low metastatic potential in both male and female mice. These findings are consistent with the results of staining for sympathetic fibers as well. Further, specifically comparing among male and female tumor nerve densities, we observed higher nerve density in tumors from female mice, for overall nerve fibers as well as sympathetic fibers.

We have also recorded the daily neural activity from breast tumors [20], where two peaks of high neural activity were observed during in-vivo tumor progression. In breast tumors as well, the neural activity was found to be diminished in 6-OHDA treated mice indicating peripheral sympathetic neural origin of the activity [20]. Thus, it can be concluded that in solid breast and melanoma tumors, sympathetic nerves are the origin of the neural activity where neural activity patterns are observed in forms of the peaks. We therefore suggest that peripheral sympathetic nerves produce neural activity with specific peak activity patterns in both the cancer types and this principle can be tested in more cancer types.

## Supporting information

S1 File (DOCX)
