# Correction: Neural recordings can differentiate between spontaneously metastasizing melanomas and melanomas with low metastatic potential

**DOI:** 10.1371/journal.pone.0318831

**Published:** 2025-01-30

**Authors:** Jay Shiralkar, Tiana Anthony, Grant A. McCallum, Dominique M. Durand

The Data Availability statement for this paper is incorrect. The correct statement is: Data are publicly available on the SPARC Portal (RRID:SCR_017041) at https://doi.org/10.26275/wlhm-9uyz.
