# Supplementary material for: Neural recordings can differentiate between spontaneously metastasizing melanomas and melanomas with low metastatic potential
Source: PLoS One. 2024 Feb 15;19(2):e0297281. doi: 10.1371/journal.pone.0297281 (PMC10868782; doi:10.1371/journal.pone.0297281)
Supplement: S1 File — (DOCX) [file pone.0297281.s001.docx]

**Supplementary Materials:**

**Neural recordings can differentiate between spontaneously metastasizing melanomas and melanomas with low metastatic potential.**

         Jay Shiralkar et. al.

^*^Corresponding Authors: [dxd6@case.edu](mailto:dxd6@case.edu), gam19@case.edu


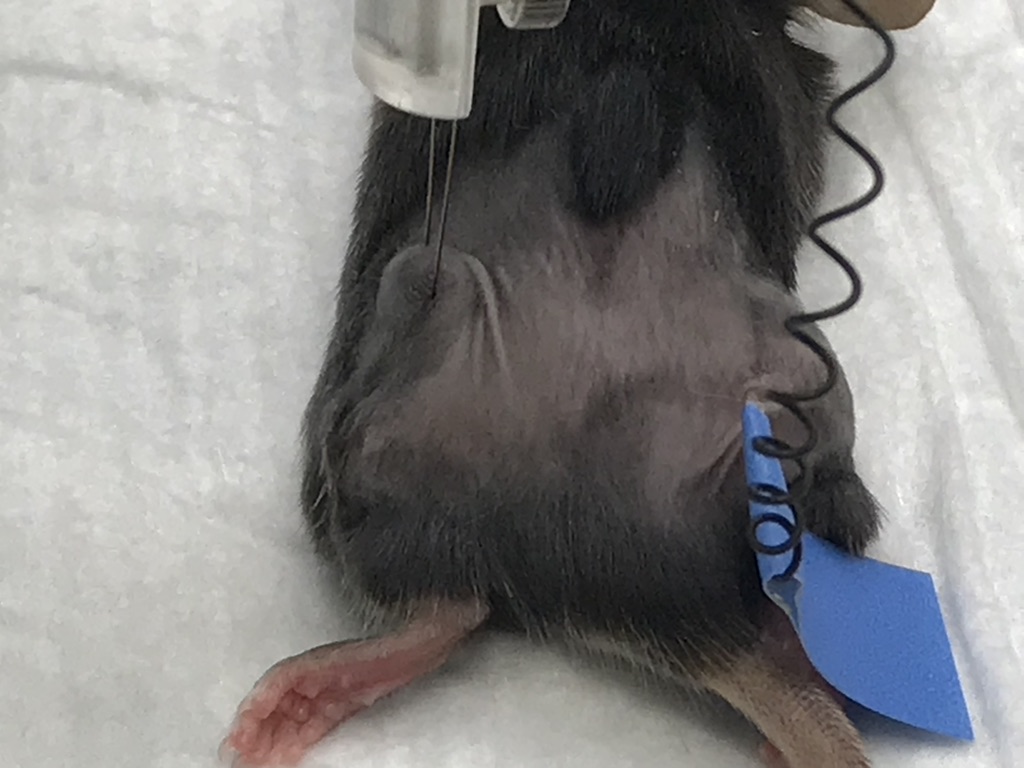


Laptop

(Data storage)

ADInstruments

amplifier

**Fig S1. Microneurography Hardware Setup.** This figure shows a simple schematic for the hardware setup used for microneurographic recordings: The microneurography pen consisted of 3 connections. The three connections included a pair of a differential probes inserted directly into the murine melanomas and a ground pin (blue marker) implanted subcutaneously below dorsal healthy skin on opposite side. Microneurography head stage collected the data and were transferred to ADInstruments amplifier. The collected data were stored in laptop using Lab Chart program.

F

E

A


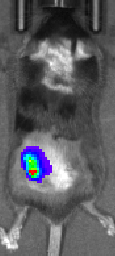

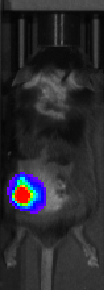

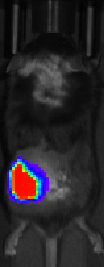

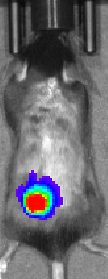

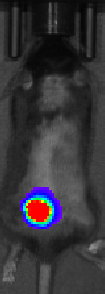

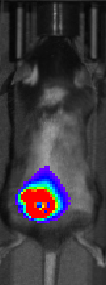

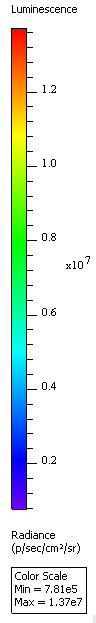


C

D

B

**Fig S2. Bioluminescent images from male and female mice bearing B16-F10 derived metastasizing tumors.** The figure shows bioluminescence images acquired on **(A)** day 6 **(B)** day 10 **(C)** day 16 respectively from male mouse bearing B16-F10 metastasizing tumors. Also, panels, **(D)** day 6 **(E)** day 10 **(F)** day 16 shows bioluminescence images acquired from female mouse bearing B16-F10 metastasizing tumors. Distant metastasis to cranial region was correlated with the neural peak activity in male and female mice.


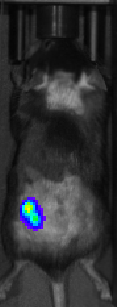

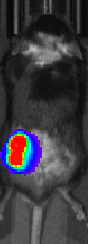

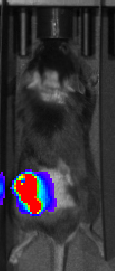

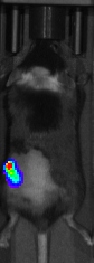

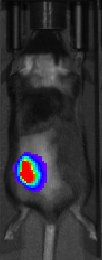

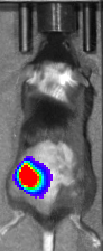

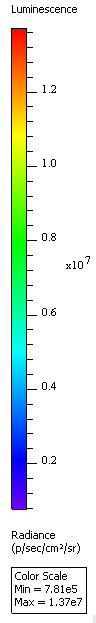


D

F

E

C

B

A

**Fig S3. Bioluminescent images from sympathectomized male and female mice bearing B16-F10 derived metastasizing tumors.** The figure shows bioluminescence images acquired on **(A)** day 6 **(B)** day 10 **(C)** day 16 respectively from male sympathectomized mouse bearing B16-F10 metastasizing tumors. Also, panels, **(D)** day 6 **(E)** day 10 **(F)** day 16 shows bioluminescence images acquired from sympathectomized female mouse bearing B16-F10 metastasizing tumors. Sympathectomy exerted sex-specific effects on distant metastasis to cranial region.

**** ****

Female

Male

**

**

****

****

p<0.05;

N=5.

p<5x10^-4^;

N=5.

B

A

Male

Female

**

**

***

***

p<0.05;

N=5.

p<5x10^-3^;

N=5.

C

D

**Fig S4. Comparison of bioluminescence flux among metastasizing and low metastatic potential bearing tumors.** We observed significantly reduced average bioluminescence flux in low metastatic potential tumor bearing mice in primary tumor mass area in (A) female mice (N=5, rank-sum test, p<5x10^-4^) as well as (B) male mice (N=5, rank-sum test, p<0.05). Similar results were observed with significantly reduced average bioluminescence flux in low metastatic potential tumor bearing mice in cranial area in (C) female mice (N=5, rank-sum test, p<5x10^-3^) as well as in (D) male mice (N=5, rank-sum test, p<0.05).

****

Male

Female

B

A

***

p<5x10^-3^;

N=5.

*****

p<5x10^-6^

N=5.

*****

***

**Fig S5. Comparison of bioluminescence flux from the primary tumor mass region among B16-F10 derived metastasizing tumor bearing mice and sympathectomized B16-F10 derived metastasizing tumor bearing mice.** The results indicate that in (A) female mice and (B) male mice i.e. in both the sexes sympathectomy caused the reduction in the average bioluminescence flux quantified from the primary tumor masses. (For males: N=5, rank-sum test, p<5x10^-3^ ; For females: N=5; rank-sum test, p<5x10^-6^).

B

A

NF

TH

*

*

p<0.05;

N=5;

n=24

p<0.05;

N=5;

n=24

*

*

**Fig S6. Sex-wise analysis of NF and TH stained primary melanoma tissues from B16-F10 metastasizing melanomas.**
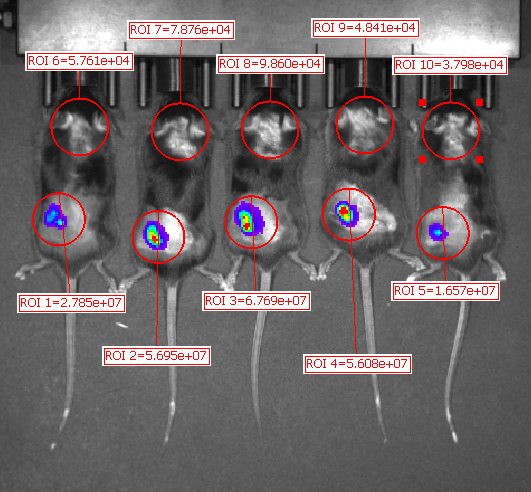
**(A)** We observed significantly higher nerve density in NF stained female tumor slices compared to male tumor slices (N=5; n=24, two-sample t-test, p<0.05). **(B)** Similarly, we observed significantly higher nerve density in TH stained female tumor slices compared to male tumor slices (N=5; n=24, two-sample t-test, p<0.05).

A

B
